# Comparaison des données de l’échographie par rapport à l’électroneuromyogramme dans le diagnostic de syndrome de canal carpien

**DOI:** 10.11604/pamj.2019.34.50.7772

**Published:** 2019-09-25

**Authors:** Siham Bouchal, Aouatef El Midaoui, Khadija Berrada, Abourazzak Fatima Zahra, Nassira Aradoini, Taoufik Harzy, Mohamed Faouzi Belahsen

**Affiliations:** 1 Service de Neurologie, CHU Hassan II, Fès, Maroc; 2 Service de Rhumatologie, CHU Hassan II, Fès, Maroc

**Keywords:** Syndrome de canal carpien, échographie, électroneuromyogramme, Carpal tunnel syndrome, echography, electroneuromyography

## Abstract

L’objectif de cette étude est d’évaluer l’utilité de l’échographie dans le diagnostic de syndrome du canal carpien par rapport à l’électroneuromyogramme (ENMG). Il s’agit d’une étude transversale incluant 50 patients sur une période de 6 mois. Tous les patients avaient bénéficié d’une échographie avec mesure de la surface du nerf médian à l’entrée du canal carpien et une étude électroneuromyographique des 2 poignets. La sensibilité et la spécificité de l’échographie ont été comparées à ceux de l’ENMG comme référence. L’âge moyen est de 49,6 ans avec une nette prédominance féminine dans 98%. La majorité des patientes étaient des femmes au foyer. Les paresthésies étaient le motif le plus fréquent de consultation dans 86%. La symptomatologie clinique était bilatérale dans 78% des cas. L’ENMG était pathologique chez 89 poignets (89%). L’échographie était anormale chez 63 poignets (63%) avec une médiane de la surface du nerf médian de 11 mm^2^. D’après cette étude, la sensibilité d’échographie était de 70%, et la spécificité était de 100% avec une valeur prédictive positive (VPP) de 100% et une valeur prédictive négative (VPN) de 29,7%. On a conclu que l’échographie n’est sensible que pour le syndrome de canal carpien avec une atteinte sévère à l’ENMG.

## Abstract (en)

This study aims to evaluate the advantages of ultrasound in the diagnosis of carpal tunnel syndrome by comparing it with electroneuromyography (ENMG). We conducted a cross-sectional study over a period of 6 months. All patients underwent ultrasound with measurement of the surface of the median nerve at the entrance of the carpal tunnel and electroneuromyographic examination of both wrists. The sensitivity and specificity of ultrasound was compared to those of ENMG. The average age of patients was 49.6 years with a clear female predominance (98%). The majority of patients were housewives. Paresthesias were the most common reason for consultation in 86%. Bilateral clinical manifestation occurred in 78% of cases. ENMG showed pathological result in 89 wrists (89%). Ultrasound was abnormal in 63 wrists (63%) with a median surface area of the median nerve of 11 mm^2^. This study highlights an ultrasound sensitivity of 70%, a specificity of 100% with a positive predictive value (PPV) of 100% and a negative predictive value (VPN) of 29.7%. It was concluded that ultrasound has sensitivity only to carpel tunnel with severe involvement on ENMG.

## Introduction

Le syndrome du canal carpien (SCC) est la mononeuropathie du membre supérieur la plus fréquente particulièrement chez la jeune femme et constitue un motif de consultation fréquent. Il est secondaire à une compression du nerf médian dans un défilé ostéofibreux appelé le canal carpien. Il est le plus souvent idiopathique mais il peut être secondaire à des causes anatomiques, traumatiques, endocriniennes, rhumatismales ou tumorales [1, 2]. L'électroneuromyogramme (ENMG) reste l’examen paraclinique de référence pour confirmer un diagnostic clinique du SCC et pour préciser sa sévérité, mais il reste un examen inconfortable pour le patient, coûteux et non disponible dans tous les hôpitaux du Maroc [2]. Au cours des dernières années, l’échographie est considérée par certaines équipes comme examen paraclinique alternatif utile pour le diagnostic du SCC, en fournissant des informations morphologiques sur le contenant (parois osseuses, et ligament annulaire) et sur le contenu (nerf médian (épaississement, modification de l’echogénicité), tendons fléchisseurs des doigts, éventuel kyste, tumeur ou corps musculaire anormal) du canal carpien permettant ainsi un diagnostic étiologique du SCC. C’est un examen anodin, confortable, rapide et plus disponible que l’ENMG [3]. L’objectif de ce travail était d’évaluer l’apport de l’échographie dans le diagnostic de syndrome de canal carpien.

## Méthodes

Il s’agit d’une étude transversale réalisée sur une période de 6 mois de juin 2014 à décembre 2014 au service des explorations fonctionnelles neurologiques au Centre Hospitalier Hassan II Fes au Maroc. Nous avons regroupés 50 patients adressés par leur médecin traitant (traumatologue, rhumatologue ou neurologue) pour suspicion de SCC. Les données ont été recueillies à l’aide d’une fiche d’exploitation préétablie qui portait notamment sur les renseignements démographiques (age, sexe, profession), les données cliniques y compris les symptômes cliniques, l’horaire de survenue, l’examen clinique, ainsi que les antécédents des patients (diabète, dysthyroidie, insuffisance rénale, hémodialyse, grossesse). Les patients ont été classés selon les critères de Katz et Stirrat: syndrome typique (fourmillements, picotements, engourdissement ou hypoesthésie avec ou sans douleur atteignent au moins deux des trois premiers doigts. La paume et le dos de la main sont exclus. Une douleur spontanée du poignet ou irradiant en remontant en direction du coude existe); syndrome probable (les signes sont identiques mais touchant aussi la face palmaire de la main, zone cubitale exclue); syndrome possible (les fourmillements, picotements, engourdissement ou hypoesthésie avec ou sans douleur atteignent au moins un doigt parmi les trois premiers); syndrome improbable (aucun symptôme n’existe dans les trois premiers doigts).

Les critères d’exclusion étaient un tableau clinique compatible avec une radiculopathie cervicale ou polyneuropathie, un antécédent de chirurgie ou d’infiltration du canal carpien ou du poignet et un antécédent de traumatisme de poignet (fractures, entorses). L’électroneuromyographie (ENMG) fut réalisé pour tous les patients par un Neurologue spécialiste en ENMG en utilisant un appareil de marque Neurosoft- MEP 4. Les paramètres étudiés étaient l’étude de la conduction nerveuse sensitive (VCS) qui se fait de façon orthodromique (température cutanée supérieure à 30°C) du nerf médian au majeur et index et du nerf ulnaire à l’auriculaire, la mesure de l’interlatence sensitive médio-ulnaire à l’annulaire (DLS4) et l’étude des conductions motrices des nerfs médian et ulnaire. Les valeurs retenues pathologiques étaient 0,5 ms pour la DLS4, la VCS est considérée comme pathologique lorsqu’elle est inférieure ou égale à 45 m/s. L’amplitude de la réponse sensitive est pathologique quand elle est inférieure ou égale à 12 microμV. La latence distale motrice (LDM) était mesurée et la valeur était pathologique si elle est supérieure ou égale à 4 m/s. L’amplitude de la réponse motrice du nerf médian est considérée comme pathologique si elle est inférieure à 6 mV. L’examen de détection est effectué sur le court abducteur du pouce à l’aide d’une aiguille concentrique monofilaire.

La sévérité de l’atteinte électrique du nerf médian a été répartie en 5 grades: négative (étude de conduction nerveuse normale: aucune preuve électrique de SCC), légère (ralentissement de la VCS avec une LDM normale), modéré (ralentissement de la VCS et allongement de LDM), sévère (absence de VCS avec LDM allongée), extrême (absence de réponses motrices et sensitives) [4, 5]. Une échographie fut proposée pour tous les patients et fut réalisée par un rhumatologue ayant un diplôme certifié d’échographie ostéoarticulaire. L’appareil utilisé était un échographe de marque Esaote type My Lab 25 Gold. L’exploration échographique du canal carpien a compris une exploration en mode B avec mesure de la surface de section du nerf médian à l’entrée du canal carpien (CC) en mm^2^ et l’étude de son échogénicité couplé à une exploration en mode Doppler. Les deux cotés ont été systématiquement étudiés. La valeur pathologique fixée de la surface de section du nerf médian à l’entrée du CC est ≥ à 10 mm^2^. Pour l’analyse statistique, toutes les variables étaient résumées par l’utilisation des statistiques descriptives. Les variables qualitatives étaient décrites en termes de proportions et les variables quantitatives étaient décrites en termes de moyenne, valeurs extrêmes et écart-type. Dans un deuxième temps, une analyse univariée a été faite pour étudier l’association entre chacune des variables explicatives et la présence du syndrome de canal carpien. Lors de la comparaison de groupes, nous avons utilisé les tests paramétriques classiques (test de Khi2, test de Student) en fonction de la nature des variables à comparer. La sensibilité, la spécificité, la valeur prédictive positive (VPP) et la valeur prédictive négative (VPN) ont été calculés pour évaluer la performance de l'échographie pour le diagnostic du syndrome de canal carpien.

## Résultats

Cinquante patients (100 poignets) ont consulté au service des explorations fonctionnelles du système nerveux pour suspicion de syndrome de canal carpien. L’âge moyen est de 49,6 ans (+/- 8) avec une nette prédominance féminine de 98 (98%). La majorité des patientes étaient des femmes au foyer 82 (83,67%). 36 patients n’avaient aucun antécédent médical, 11 patients étaient diabétiques et 3 patients avaient une dysthyroïdie. Les paresthésies étaient la plainte principale des patients (86 poignets soit 86%) [77,63%-92,3%]. La douleur était présente dans 52 poignets (52%) [41,78% -62,10%]. L’existence des 2 symptômes a été notée dans 52 poignets (52%). L’horaire de la survenue des symptômes était nocturne chez 27 patients (54%) et mixte chez 21 patients (42%) et matinales uniquement chez 2 patients. La symptomatologie clinique était bilatérale chez 39 patients (78%). L’examen clinique a révélé une atteinte motrice clinique pour 12 poignets (12%) [6,36%-20,02%] et une atteinte sensitive dans 32 poignets (32%) [23,02%; 42,08%]. Le signe de Tinnel est positif dans 55 poignets (55%) et le signe de Phalen dans 40 poignets (40%). La plupart des patients consultent tardivement avec une durée moyenne d’évolution des symptômes de 51 mois. L’ENMG était anormal chez 89 poignets (89%); l’atteinte était légère chez 40 patients, modérée chez 28 patients et sévère chez 21 patients; aucune atteinte extrême n’a été notée. L’échographie était anormale chez 63 poignets (63%) avec une médiane de la surface du nerf médian de 11 mm^2^ avec des extrêmes de 4 et 23 mm^2^ (Tableau 1). L’échogénicité était pathologique dans 13 poignets (13%). D’après cette étude, la sensibilité d’échographie était de 70%, et sa spécificité était de 100% avec une valeur prédictif positif (VPP) de 100% et une valeur prédictif négatif de 29,7%. D’après le Tableau 1, plus l’atteinte du nerf médian est sévère à l’ENMG plus l’échographie devient très sensible. L’échographie était pathologique dans 95,3% lorsque l’atteinte était sévère à l’ENMG.

**Tableau 1:** Les données de l’échographie et celles d’électroneuromyogramme (ENMG)

Echographie	Atteinte à l’ENMG			
	Normale	Légère	Modérée	Sévère
Normale	11(100)	19(47,5)	6(21,4)	1(4,8)
Pathologique	0(0)	21(52,5)	22(78,6)	20(95,3)

## Discussion

Le diagnostic de SCC est principalement clinique basé sur l'interrogatoire et l’examen clinique [6, 7]. Il existe plusieurs classifications cliniques dont leur utilisation en pratique courante est différente entre les praticiens. Le symptôme le plus fréquent est les paresthésies dans le territoire du nerf médian comme c’est le cas dans notre série [6]. Le recours à l’ENMG est devenu incontournable pour confirmer l’atteinte du nerf médian au poignet et surtout lorsqu’un traitement chirurgical est envisagé. L’ENMG permet aussi d’évaluer la sévérité de cette atteinte et d’en préciser le niveau. Il contribue également à éliminer d’autres pathologies mimant un SCC comme les névralgies cervico-brachiales [8-10]. Cependant, cet examen reste inconfortable, couteux, non disponible dans la plupart des hôpitaux au Maroc. L’échographie, méthode moderne, cherche sa voie dans l’exploration du SCC et pour évaluer sa sensibilité, sa spécificité et son utilité de l’échographie dans le diagnostic de SCC, plusieurs études ont été décrites ces dernières années et se sont intéressés à la comparaison entre des anomalies morphologiques à l’échographie et physiologiques à l’électroneuromyogramme (ENMG).

Une méta-analyse évaluant la sensibilité et la spécificité de l’échographie dans le diagnostic du SCC publiée en 2006, a rapporté que la sensibilité varie de 67 à 89%, et la spécificité varie de 57 à 97%. Elle a expliqué cette grande variabilité des valeurs par le choix différent de référence pour définir le SCC qui a été la clinique associée à un ENMG anormal dans neuf études sur 11, par la taille des échantillons, et par la variabilité des valeurs normales et pathologiques fixées [7]. Les valeurs de la sensibilité et la spécificité de notre travail sont inclus dans les intervalles rapportés dans cette méta-analyse mais on avait un taux de faux négatif qui était élevé. Cette méta-analyse a conclu aussi que l’échographie ne peut pas remplacer l’ENMG puisqu’elle ne met pas en évidence une anomalie évocatrice d’une compression du nerf médian au poignet que dans 55% des cas alors que l’ENMG peut en détecter dans plus de 90% [7], et que même sur le plan économique, l’ENMG, s’il se limitait comme l’échographie à détecter une anomalie du nerf médian au poignet aurait un coût et un temps de réalisation équivalent à ceux de l’échographie. Par contre, l’échographie autant qu’un examen morphologique permet d’éliminer un SCC secondaire à une cause locale comme un kyste, une synovite, une tumeur, un anévrysme; donc pour cette méta-analyse, il y a une complémentarité entre l’ENMG et l’échographie et pas une concurrence [7]. L’étude de Kwon *et al.* publiée en 2008 a comparée la sensibilité et la spécificité de l’échographie et ceux de l’ENMG en prenant la clinique comme référence. La sensibilité était similaire entre l'échographie et l’ENMG (p = 0,27), alors que la spécificité était significativement plus faible en échographie que l’ENMG (p = 0,02). Il a conclu que l’échographie n’était pas assez précise pour remplacer l’ENMG. Cependant, les auteurs ont critiqué leur étude par le choix des critères clinique trop stricts de SCC [1].

Une autre méta-analyse publiée en 2011 de 19 articles parmi 323 articles avec un échantillon de 3131 poignets, la sensibilité et la spécificité de l'échographie dans le diagnostic du SCC est de 77.6% [71,6%-83,6%] et 86,8% [78.9%-94,8%] respectivement. Vu ces grandes variations aussi bien de la sensibilité que de la spécificité rapportées dans les études concernées, on n’a pas pu conclure que l'échographie est un outil de confirmation de SCC mais une alternative possible [10]. Notre étude a montrée que la mesure de la surface du nerf médian par l’échographie à l'entrée du canal carpien peut être une alternative à l’ENMG dans les atteintes sévères. Donc l’ENMG est fortement indiqué en cas de suspicion clinique de SCC avec une échographie normale vu sa valeur prédictive négative très basse. Cette conclusion est comparable à celle d’une étude faite par Moran *et al.* [11]. Cependant, notre étude a des limites qui sont: l’exploration des 2 poignets même le coté asymptomatique et l’utilisation de poignets asymptomatiques de patients souffrant d’un SCC d’un seul coté comme poignet témoin qui reste discutable car une atteinte controlatérale infra-clinique n’est pas rare; l’absence de groupe témoin et la taille petite de l’échantillon constitue également un point faible de notre étude.

## Conclusion

L’échographie du canal carpien ne permet pas de diagnostiquer l’ensemble des cas de syndrome de canal carpien avec un ENMG pathologique et donc elle ne peut pas remplacer l’ENMG au vu de sa sensibilité modérée et sa faible valeur prédictif négatif. Cependant et vue les limites de ce travail, une étude cas-témoin et multicentrique reste souhaitable avec un échantillon plus large pour confirmer ou affirmer ces résultats.

### État des connaissances actuelles sur le sujet

- L’ENMG est une exploration incontournable pour confirmer le diagnostic de SCC et sa sévérité, mais c’est un examen inconfortable, coûteux, non disponible dans la plupart des hôpitaux.

### Contribution de notre étude à la connaissance

- Évaluer la sensibilité, la spécificité et l’utilité de l’échographie dans le diagnostic de SCC par rapport à l’ENMG, puisque l’échographie reste plus confortable et plus disponible.

## Conflits d’intérêts

Les auteurs ne déclarent aucun conflit d'intérêts.
